# Progress in the PRIDE technique for rapidly comparing protein three-dimensional structures

**DOI:** 10.1186/1756-0500-1-44

**Published:** 2008-07-11

**Authors:** Svetlana Kirillova, Oliviero Carugo

**Affiliations:** 1 Department of Biomolecular Structural Chemistry, Programme of Structural and Computational Biology, Max F. Perutz Laboratories, Vienna University, Campus Vienna Biocenter 5, A-1030 Vienna, Austria; 2 Department of General Chemistry, Pavia University, Viele Taramelli 12, I-27100 Pavia, Italy

## Abstract

**Background:**

Accurate and fast tools for comparing protein three-dimensional structures are necessary to scan and analyze large data sets.

**Findings:**

The method described here is not only very fast but it is also reasonable precise, as it is shown by using the CATH database as a test set. Its rapidity depends on the fact that the protein structure is represented by vectors that monitors the distribution of the inter-residue distances within the protein core and the structure of which is optimized with the Freedman-Diaconis rule.

**Conclusion:**

The similarity score is based on a *χ*^2 ^test, the probability density function of which can be accurately estimated.

## Findings

Although numerous methods for comparison protein three-dimensional (3D) structures were designed, we still lack a unique, commonly accepted procedure to measure the structural diversity between proteins [1]. In particular, the structures of distantly related proteins should be expressed by the appropriate way allowing their comparison and the 3D structure representations used in modern algorithms are described in the reviews [2,3]. The most accurate protein structure comparison methods produce protein structure alignments that are computationally intensive. Slower techniques may be preferable to analyze and classify sufficiently small data sets. However, the time criterion is crucial in the case of integrated survey of large databases, like the Protein Data Bank or the domain collections CATH and SCOP [4]. This problem is very similar to that encountered few years ago in the case of macromolecular sequence databases, which was solved by the development of tools like FASTA [5], BLAST [6] or PSI-BLAST [7] that allow one to effectively scan enormous databases like UniProt [8], which presently contain several millions of entries. Although protein 3D structure databases are still much smaller, several representations of protein structure suitable for rapid comparison without alignment were proposed [9-13]. One of the fast and automatic techniques for protein structural comparison is PRIDE [9]. In this method the protein structure is represented via a series of distributions of inter-atomic distances allowing the use rapid comparison procedure without alignment.

In the present communication, some improvements of the original PRIDE technology are presented. They make it more accurate than the original version without decreasing its speed. The classification ability of the method was tested on the CATH database.

### The PRIDE methodology

In original PRIDE version, a protein structure in defined by the distributions of the distances between C_*α*i _and C_*α*(i+n) _atoms, where n, which ranges from 3 to 30, is the number of C_*α *_atoms between them in the backbone joint. The comparison between two protein 3D structures is reduced to the comparison between distributions of inter-residue distances. This is performed by chi-square contingency table analysis, which estimates whether two distributions represent the same overall population and allows one to compute a probability of identity P, ranging from 0 and 1. Since 28 pairs of histograms are compares, 28 P values are obtained and then averaged to give the overall PRobability of IDEntity (PRIDE) between the two protein 3D structures. Such a similarity score can range, by definition, from 0 to 1, the latter value indicating the identity between the two protein structures. In the next sections, four modifications, introduced into this computational procedure, will be described.

### Amount of structural information

The maximal value of n, which was equal to 30 in the old PRIDE version, is now selected as a function of the protein dimension. Obviously, the histograms, in which inter-residue distances are binned, must have a sufficiently high number of observations to be compared via any statistical tool. The number of observations in the histograms increases with the length of the protein and decreases with n. Therefore, histograms were generated for all n values larger than 3 and lower than n_max_, where n_max _is the value for which there are only 20 C_*α*i_-C_*α*(i+n) _distances. Clearly, if n > n_max_, the histograms would contain less than 20 observations and they were thus ignored. Therefore, the numbers of histograms are different for proteins of different length in the modified PRIDE version. In the comparison of two domains, represented by series of C_*α*i_-C_*α*(i+n) _histograms, with 3 ≤ n ≤ n_max1 _for the first domain and 3 ≤ n ≤ n_max2 _for the second domain, the maximal value of n (n_max_) was defined as

*n*_max _= min(*n*_max1_, *n*_max2_)

Moreover, only distances between residues belonging to helices and/or strands were taken into account in the modified PRIDE version, in order to increase the computational speed of the method. The STRIDE package, based on the detection of hydrogen bonds patterns and backbone torsions, was used for secondary structure assignment [14].

### Optimization of the dimension of the histogram intervals

The building of a regular histogram from continuous data demands a cautious specification of the number of bins. In the old version of PRIDE, each bin width was arbitrarily set to 0.5 Å, and adjacent bins were merged together so that at least 5% of the observations were included in each bin. Here a more rigorous approach was followed. Firstly, inter-residue distances were binned in the histograms with a fixed bin width of 0.1 Å, a value close to the average expected uncertainty of protein atomic coordinates obtained with crystallographic methods [15]. Then bin widths are changed automatically to their optimal value BS by using the Freedman-Diaconis rule [16]

*BS *= 2*iqr*(*x*)*k*^-1/3^

where k is the number of observations in the sample x; iqr(x) is the interquartile range of the data of sample x, that is the range between the third and first quartiles. The iqr is expected to include about half of the data. The optimal BS values are computed for a query protein structure, and then they are used to change the histogram bins for all domains in the scanned database. New optimal BS values must be recomputed for a new query. Despite this might seem to be rather complicated and time consuming, we verified that once the histograms for the entire database are pre-computer and stored with very small bins of 0.1 Å, all of them can be re-shaped to the optimal BS very rapidly (see the paragraph "Computational speed" below).

### Distribution comparisons

While in the original version of PRIDE, the C_*α*i_-C_*α*(i+n) _distance distributions were compared using the contingency tables [17], another statistical procedure is applied now. Contingency tables are more suitable to analyze relationships between nominal (categorical) variables and can be applied to compare continuous distributions only by carefully selecting an arbitrary bin size in such a way that each bin contains sufficient data. Here we adopted another approach that is more suitable to compare continuous distributions and that is computationally not more demanding than the contingency table analysis. By assuming that the distributions of both binned data sets of inter-residue distances are equally unknown, it is possible to use the chi-square test to disprove the null hypothesis that the two data sets can be described by the same distribution. If R_i _is the number of observations in bin i for the first protein and S_i _is the number of observations in the same bin i for the second protein, then the chi-square statistics is

$$\chi^{2}=\sum_{i}\frac{{\left(R_{i}\sqrt{\frac{S}{R}}-S_{i}\sqrt{\frac{R}{S}}\right)}^{2}}{R_{i}+S_{i}}$$

where

$$R=\sum_{i}R_{i}$$

and

$$S=\sum_{i}S_{i}.$$

*χ*^2 ^ranges from 0 to the positive infinity. A large value of *χ*^2 ^indicates that the null hypothesis is rather unlikely and that the two proteins are considerably different, and *χ*^2 ^can thus be used as a statistical measure of proximity between two protein 3D structures. On the contrary, two identical protein 3D models are associated with a *χ*^2 ^value equal to 0.

Furthermore, the degree of proximity between two protein structures can be also expressed by an incomplete gamma function determining the chi-square probability density function:

$$P(N_{b},\chi^{2})=1-\frac{\Gamma (N_{b},\chi^{2})}{\Gamma (N_{b})}=\frac{1}{\Gamma (N_{b})}\int_{\chi^{2}}^{\infty }e^{-t}t^{N_{b}-1}dt$$

where N_b _is the number of histogram bins, that corresponds to a number of degrees of freedom for histograms with an unequal number of observations. In this case the proximity measure P ranges from 0 to 1 corresponding, respectively, to the completely different and to the identical protein folds.

$$\chi_{n}^{2}$$

and P_n _are computed for each pair of histograms of the C_*α*i_-C_*α*(i+n) _distances for 3 = n = n_max_. Then they are averaged to estimate the global degree of protein structural proximity. It must be observed that while *χ*^2 ^is a distance measure of proximity, with lower values associated with two domains that are similar, P is a measure of similarity, with higher values associated with two domains that are similar. Beside this difference, both can be used as structural similarity scores and monitor exactly the same protein structural features. However, P has the definite lowest and highest limits that are equivalent to the similarity score used in the old PRIDE version.

### Computational speed

Given the extreme simplicity of the algorithm, it is not surprising that computations can be very fast. The most time consuming step is the computation of the histograms of the C_*α*i_-C_*α*(i+n) _distributions. However, they can be pre-computed and stored in about 850 seconds (Xenon 3 GHz processor) for the 34,035 protein domains of Table 1, 29,098 of which are long enough to be represented by at least 30 histograms and 4,937 of which are smaller and can be represented by 10–30 histograms. The comparison of a query with all the database entries takes on average 170 seconds (by using all the queries of Table 1), 20 of which are needed for the optimization of the bin size, according to the Freedman-Diaconis rule. The overall speed is nearly identical to the speed of the old PRIDE version. By comparison, the same amount of computations can be performed in about 4,000 seconds by using the SHEBA downloaded software [18]. Other computer programs, like for example VAST [18], are available only as web-servers and it is thus impossible to compare their computational speed with that of the new PRIDE version. However, it was observed the VAST server is not particularly fast [19], though this does not demonstrate that the VAST algorithm is not.

**Table 1:** The content of the datasets and the query lists used for PRIDE testing

Dataset	Number of domains in the dataset	Number of histograms used for the domain structure representation	Number of domains in the query list						
			
			E*			D**			Total
				
			*α*	*β*	*α*/*β*	*α*	*β*	*α*/*β*	
1	29 098	> 30	24	25	25	25	25	25	149
2	4 937	10 – 30	6	6	6	8	8	8	42

*E corresponds to the "easy" cases when the queries belong to highly populated groups of investigated datasets containing at least 50 domains at the homologous superfamily classification level of CATH;

**D corresponds to the "difficult cases" when queries belonged to small groups having no more than 3 domains at the homologous superfamily classification level of CATH

### Data sets

The new structure comparison method was benchmarked against the CATH v3.0.0 database [20], which is a hierarchical classification of protein domains according to the class C (prevalence of secondary structural types), architecture A (the number, type, and reciprocal orientation of the secondary structural elements), topology T (the topological connection of the secondary structural elements) and homologous superfamily H (a common evolutionary origin supported either by significant sequence similarity or significant structural and functional similarity). Two datasets were created (Table 1), one with domains large enough to be represented by at least 30 distributions of C_*α*i_-C_*α*(i+n) _distances, and the other with smaller domains, for which 10 < n_max _< 30. Domains containing more then one polypeptide chain were disregarded since, by definition, PRIDE cannot handle them.

### Query lists

A non-redundant series of CATH entries were randomly selected from different superfamilies to be used as queries, by ensuring that all the three principal classes C of the database are equally represented (Table 1). Some were large domains (n_max _> 30) and other small domains (10 < n_max _< 30). About half of them were considered to be "easy" queries, in the sense that they belong to a CATH fold cluster containing at least 50 domains, and the others were "difficult" queries that belong to small CATH fold groups having no more than 3 domains.

### Performance evaluation

The performance of the new PRIDE version can be examined by the computation and the analysis of the ROC curves. The P value, which is a similarity score, is used to calculate ROC curve in the present study. A threshold similarity is consecutively decreased, with subsequent decrements equal to 0.01, in the entire range of possible P values, from 1 to 0. At each step, each of the queries (Table 1) was compared to all the entries of the databases (Table 1). As a consequence, 4,335,602 comparisons were performed by considering the dataset of large protein domains and 207,354 comparisons were necessary by considering the dataset of small protein domains.

Each comparison can be classified in one of four categories, according to the CATH classification of two domains and their P value. It can be i) a true positive (TP), if the similarity between the query and the entry is higher that the threshold value and if the query and the entry belong to the same CATH fold; ii) false positive (FP) if the similarity between the query and the entry is higher that the threshold value despite the fact that they have different CATH classification; iii) a false negative (FN), if the entry and the query are in the same fold cluster despite their estimated similarity is lower than the threshold value; iv) a true negative (TN), if the similarity is estimated to be smaller that the threshold value and if the query and the entry are actually classified into different CATH fold groups. On the basis of these definitions it is possible to compute, for each threshold value, the sensitivity and the specificity

*Sensitivity *= *TP*/(*TP *+ *FN*)

**Figure 1:**
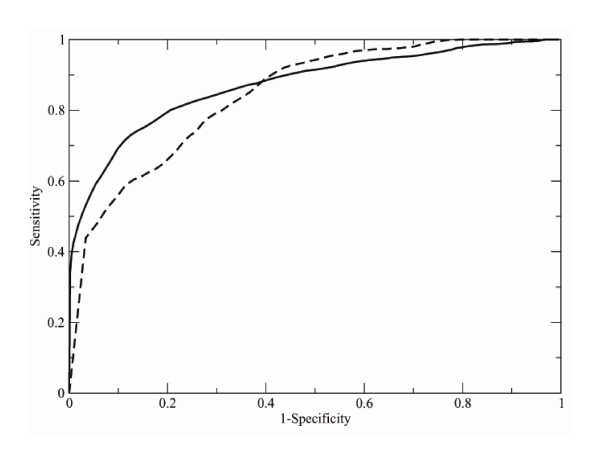
**ROC curves**. The solid line shows a ROC curve obtained by comparing 149 CATH domains with 29 098 CATH entries of the first dataset of Table 1 that contains large protein domains; the dashed line represents a ROC curve calculated for the 42 small CATH domains and 4 937 CATH entries of the second dataset of Table 1, containing small protein domains.

*Specificity *= *TN*/(*TN *+ *FP*)

and the ROC curve is obtained by potting Sensitivity against (1-Specificity) for the entire range of possible threshold values. Figure 1 shows the ROC curves obtained as described above. It is necessary to remember that the line through the origin with slope 1, that is the diagonal, would correspond to the similarity detection based on a random measure. Therefore, the area under ROC curve equal to 0.5 is related to a random similarity measure, larger values indicate better than random estimations, and a value equal to 1 indicates perfect similarity. The areas under the ROC curves, shown in Figure 1, are 0.87 and 0.82 for the first and second datasets of Table 1, respectively. Not surprisingly, the area under the ROC curve is larger (0.87) for the first dataset of Table 1, which contains larger protein domains that can be described with at least 30 histograms of C_*α*i_-C_*α*(i+n) _distances, and smaller (0.82) for the second dataset, which contains smaller proteins that are represented by a lower number of histograms. Such values are considerably better than that obtained by using the old version of PRIDE (0.55). These values are also comparable to those obtained with two other procedures for evaluating protein structure similarity – SHEBA (0.93) and VAST (0.90) that are computationally much more demanding then the methods described in the present manuscript [18]. The areas under the ROC curves were also computed by using separately queries that are classified into the *α*, *β*, and *α*/*β *classes within the CATH database in order to estimate the performance of PRIDE on different types of proteins. Values of 0.90, 0.90, and 0.83 were obtained by scanning the database of 29,098 domains with the query sets containing 49 *α *proteins, 50 *β *proteins, and 50 *α*/*β *proteins (dataset number 1 of Table 1), indicating that proteins containing both helices and strands are more difficult to be correctly identified, probably because of the higher structural diversity of protein domains containing different types of secondary structural elements. Additional information is available at [21] (Downloads section).

## Competing interests

The authors declare that they have no competing interests.

## Authors' contributions

OC supervised and coordinated the project. SK developed the algorithm, carried out the analyses, and prepared, with OC, in the writing of the manuscript. All authors read and approved the manuscript.
